# Changes in Arthrogenic Stiffness Associated with Prolonged Joint Immobilization

**DOI:** 10.1298/ptr.26-E00007

**Published:** 2026-06-20

**Authors:** Masanori WATANABE, Satoshi KOJIMA

**Affiliations:** 1 Faculty of Rehabilitation Sciences, Nagoya Gakuin University, Japan; 2 Course of Rehabilitation, Graduate School of Rehabilitation, Kinjo University, Japan

**Keywords:** Contracture, Joint stiffness, Torque curve, Rat

## Abstract

**Objectives:**

This study aimed to investigate how changes in torque curves and histological alterations in joint structures occur in relation to the duration of immobilization, using a rat knee joint contracture model.

**Methods:**

A total of 58 male Wistar rats were divided into 6 groups: 3 immobilization groups with knee joints immobilized for 4, 8, or 16 weeks and 3 control groups receiving only standard housing until the same age. After each immobilization period, the torque curves during knee extension were measured. From the torque curves, the knee extension angle at 10, 30, 60, and 120 mNm and the slope of the linear region were calculated and compared among groups. Subsequently, the articular complex structures were histologically examined. The length of the posterior joint capsule and the flatness of the tibial articular surface were calculated from tissue images and compared among groups.

**Results:**

As the immobilization duration increased, the knee extension angle significantly decreased at all torque values, and the slope of the linear region became significantly gentler. The length of the posterior joint capsule was significantly shorter in the immobilized groups than in the control groups. Additionally, the tibial articular surface was significantly flatter in the immobilized groups than in the control groups. However, no significant differences were observed between the immobilized groups.

**Conclusions:**

With joint immobilization, the shape of the torque curve changes, which is influenced by the posterior joint capsule and other factors.

## Introduction

Joint contracture is a common problem encountered in rehabilitation settings. Regardless of the location of the factors impeding joint movement, any state in which normal range of motion (ROM) is restricted is generally defined as joint contracture^1)^. Numerous findings from experimental animal studies on joint contracture indicate that joint immobilization induces restricted ROM, which worsens with prolonged immobilization^2–7)^. This restriction is mainly attributed to muscular factors during the early phase of immobilization^2–4,7)^. After 4 weeks of immobilization, arthrogenic changes become the predominant factor^2–4,7)^, and shortening and fibrosis of the posterior joint capsule occur^6,8,9)^. Although quantitative changes in ROM have been documented, qualitative changes, particularly alterations in resistance (stiffness) during joint movement, remain insufficiently investigated. Clarifying alterations in stiffness associated with joint immobilization and their underlying mechanisms may enhance our understanding of the pathophysiology of joint contracture and provide new insights into its management. If the importance of qualitative improvement is established, this may not only guide the selection and comparison of existing interventions, such as simple stretching, stretching combined with mobilization, and stretching with thermal modalities, but also facilitate the development of novel therapeutic approaches.

Stiffness reflects the extent of displacement in response to load, which can be measured by plotting a load–displacement curve. The initial portion of the load–displacement curve exhibits a toe region where the increase in load is small relative to the increase in displacement. Then, the curve transitions to a linear region where displacement and load increase linearly. Continued loading beyond this point leads to a failure region with a sharp decrease in load relative to displacement^10)^. The load–displacement curve in joint motion is represented as a torque–angle curve, hereafter referred to as a “torque curve.” Recent studies on joint stiffness using a model in which trauma was applied to the rat elbow joint, followed by immobilization, and using a model in which hyperextension injury was inflicted on the rabbit knee joint, followed by immobilization, have shown that immobilization increases the torque early in the joint motion and shifts the torque curve to a narrower ROM^11,12)^. However, these studies provided inconsistent descriptions of changes in the shape of the torque curve, such as differences in the slope of the linear region. Furthermore, to the best of our knowledge, no study using a simple immobilization model has verified the torque curve of the joint complex, leaving this aspect unclear.

Immobilization models involving joint component damage can cause more severe contractures^13)^. A simple immobilization model that excludes inflammatory effects is essential for simulating immobilization associated with plaster casts or prolonged bed rest. Therefore, this study aimed to clarify changes in torque curves and histological alterations in joint components over the immobilization period using a rat knee joint contracture model, in which the joint components were immobilized for 4–16 weeks without injury.

## Methods

All procedures were conducted in accordance with the Nagoya Gakuin University Animal Experiment Regulations and approved by the university’s Animal Experiment Committee (Approval No. 2021-001). A total of 48 male Wistar rats aged 8 weeks (185–230 g) and 10 retired rats (approximately 24 weeks old, 375–415 g) were included in this study. Rats were randomly divided into an immobilization group (n = 28) and a control group (n = 20). The immobilization group was further divided into 3 subgroups that underwent knee joint immobilization for 4, 8, or 16 weeks: the 4-week immobilization (Im4W) group (n = 9), the 8-week immobilization (Im8W) group (n = 10), and the 16-week immobilization (Im16W) group (n = 9). The control group was divided into 2 subgroups that underwent standard housing for the same periods: the 4-week control (Con4W) group (n = 10) and the 8-week control (Con8W) group (n = 10). The retired rats (n = 10) were treated as the 16-week control group (Con16W) (Fig. 1). All rats were housed in plastic cages with free access to food and water. Lighting was regulated on a 12-h light–dark cycle to maintain a regular circadian rhythm. The housing temperature was maintained at 25°C to minimize diurnal variation.

**Fig. 1.**
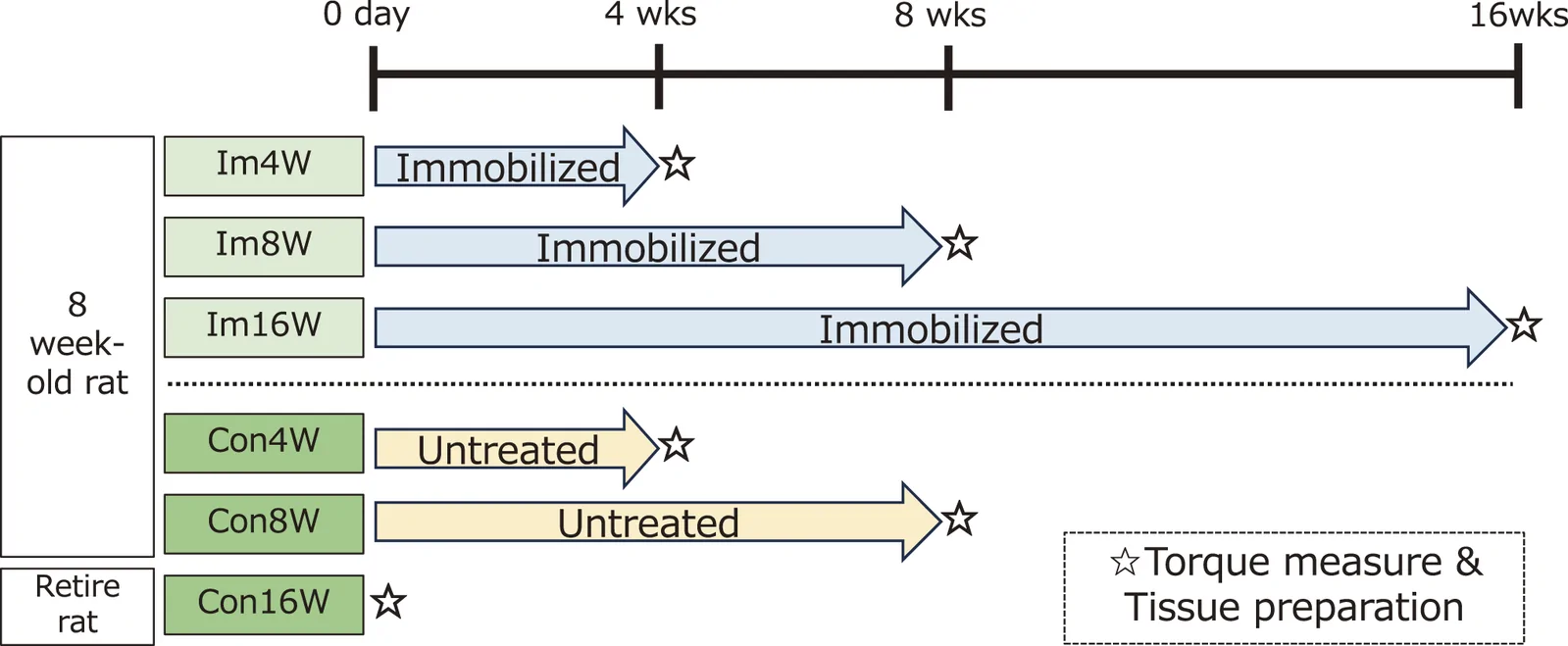
Protocols and ages for each group. The control groups were age-matched to their respective immobilization groups. The retired rats were generally 24 weeks old. Im4W, the 4-week immobilization; Im8W, the 8-week immobilization; Im16W, the 16-week immobilization; Con4W, the 4-week control; Con8W, the 8-week control; Con16W, the 16-week control; wk, week

Knee joint immobilization was performed through internal fixation using plates, as described previously^14)^. Pilot holes were drilled into the lateral aspects of the right tibia and right femur using 0.8-mm Kirschner wires (MIZUHO, Tokyo, Japan) under isoflurane inhalation anesthesia. A 1.0-mm screw was then used to secure a plastic plate, which was passed under the biceps femoris muscle, positioning the knee joint at approximately 135° of flexion.

After the rearing period, joint torque during knee extension was measured in 5–6 animals per group using a small animal exercise device^15)^ (NDH-1; Bio Research Center, Nagoya, Japan) with a custom-made knee attachment (Fig. 2). After euthanasia by isoflurane overdose, the skin covering the entire hindlimb was excised, and all posterior muscles attached to the knee joint, including hamstrings, adductor muscles, gastrocnemius, and plantaris, were immediately dissected. The lateral malleolus and fibular head were marked as anatomical landmarks to define the lower leg axis. Rats were then placed on the measurement apparatus, with the right lateral epicondyle aligned with the motor’s rotational axis and the femur fixed. The lower leg was moved in the knee extension direction at 3°/s, and the torque required during this movement was measured. Based on a previous study^16)^, the maximum torque intensity was set to 120 mNm. Video footage was captured during torque measurement from directly above using a digital video camera. Still images were extracted from the video at 200-ms intervals using the motion capture software (Kinovea, https://www.kinovea.org/) version 0.9.5. These images were used to measure the knee joint extension angle. Measurements were performed using the image editing software Photoshop (Adobe Systems, San Jose, CA, USA). The angle formed between the femoral and fibular long axes was measured. Full knee joint extension was defined as the state where the fibula reached the extension line of the femoral long axis at 180°. The torque values reported during rat knee joint ROM measurements vary widely from 12 to 160 mNm^2,3,17–19)^. Based on preliminary experiments confirming that the knee joints of normal adult rats can be hyperextended, the maximum torque in this study was set at 120 mNm. A previous study showed that knee joint stretching interventions were performed at approximately 50 mNm^19)^. Therefore, 30 mNm was selected as the standard reference for routine angle measurement, and 60 mNm was selected as the reference for maximum force application without causing tissue damage. In addition to these torque values, a lower torque level of 10 mNm, as described below, was included, and knee extension angles were compared at 4 torque levels: 10, 30, 60, and 120 mNm.

**Fig. 2.**
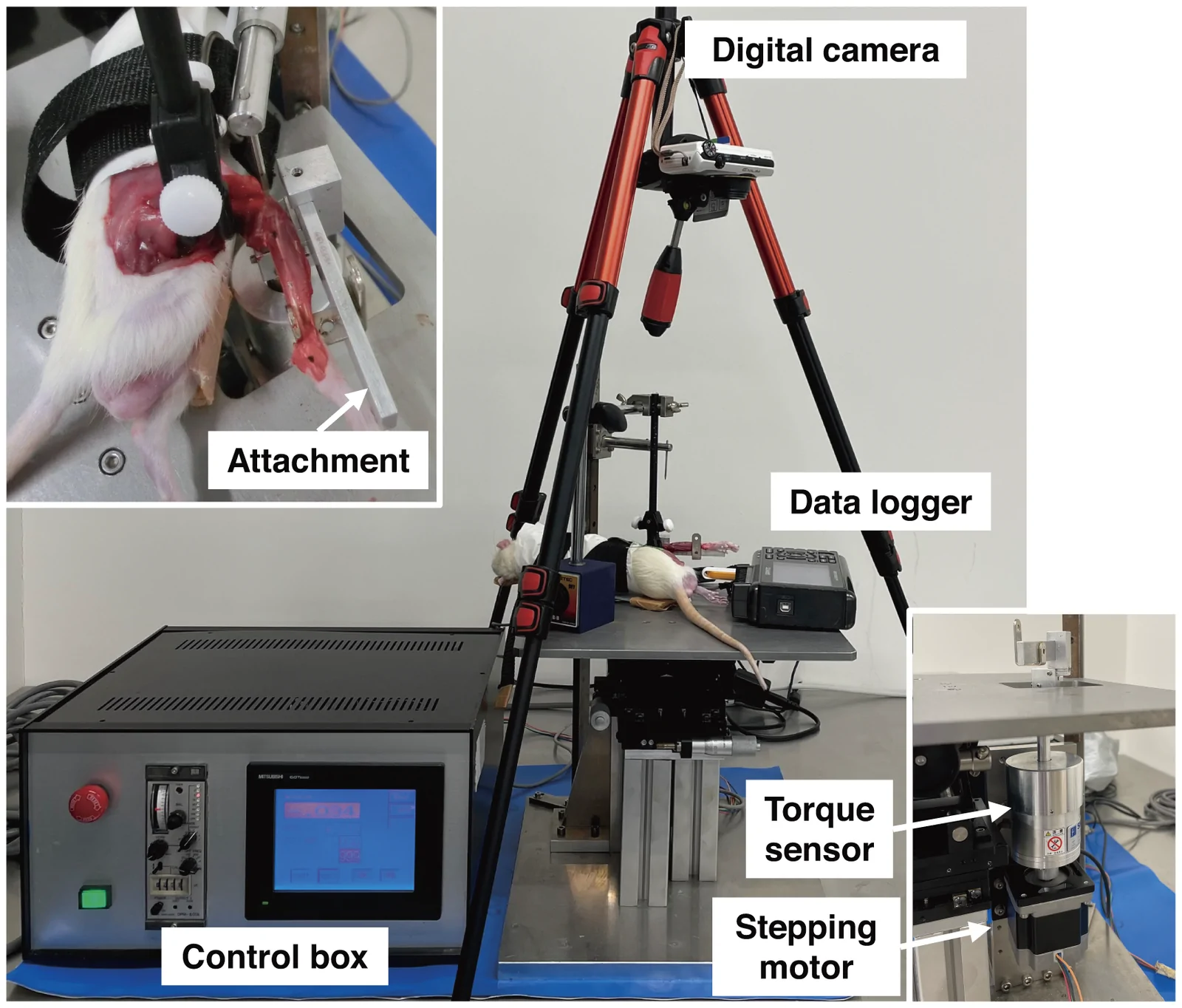
Torque measuring device. The upper-left panel shows an enlarged view of the stage, and the lower-right panel shows a side view of the stage.

A torque–angle curve was constructed using the obtained data, with torque values on the vertical axis and knee joint extension angle on the horizontal axis. The slope of the linear region was calculated by determining the best-fit line using the least-squares method. Based on a preliminarily obtained torque–angle curve of the normal knee joint (Fig. 3), the point at which the torque required for joint motion exceeded 10 mNm was uniformly defined as the onset of the linear region in both groups, although the corresponding value in the immobilized group might be lower, and the region preceding this point was defined as the toe region.

**Fig. 3.**
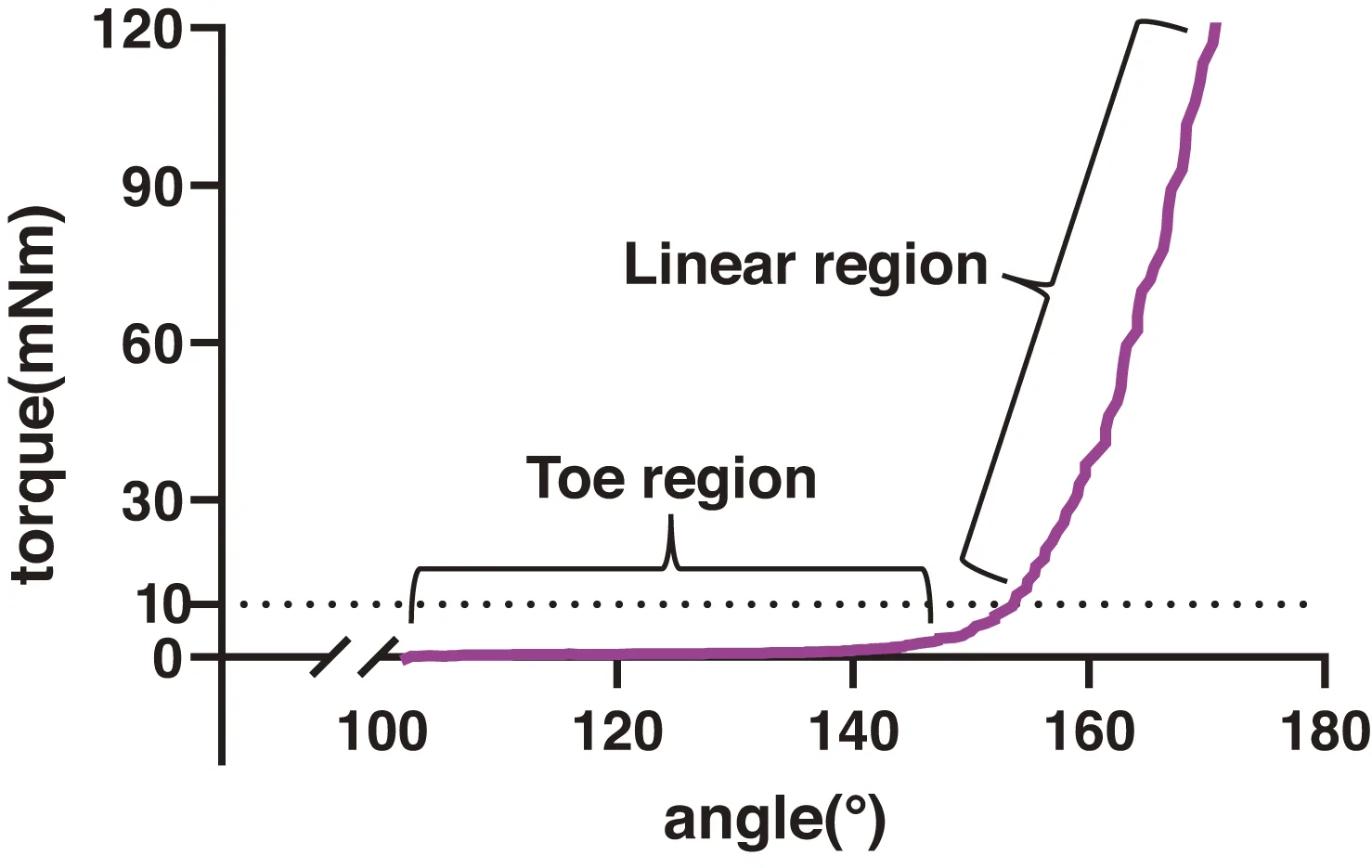
Sample torque curve (of the normal rat knee joint). The toe region is the region where the displacement per unit torque is large, whereas the linear region is the region where the torque and displacement vary linearly. A torque of 10 mNm was used as the boundary between each region. The slope of the linear region was calculated using the least-squares method.

The remaining 4 animals in each group were used for histological examination. Perfusion fixation was performed using 4% paraformaldehyde under isoflurane inhalation anesthesia, and the knee joints were harvested. After immersion fixation in the same solution for 72 h, the knee joints were decalcified in Plank–Rychlo solution for 72 h and bisected sagittally. After 72 h of neutralization, the specimens were embedded in paraffin, and 3–5-μm-thick sections were prepared using a microtome and stained with hematoxylin and eosin. The joint structure was examined using a microscope digital camera system (DP-73; Olympus, Tokyo, Japan). The length of the posterior joint capsule was measured from images acquired at ×40 magnification using ImageJ (https://imagej.net/ij/docs/faqs.html)^20)^, according to a previous study^21)^. Additionally, the flatness of the tibial articular surface was quantified from images acquired at ×20 magnification. Specifically, the XY coordinates of the cartilage surface within the range from the anterior to the posterior meniscus were obtained using ImageJ. Principal component analysis was applied to these centered coordinates. The flatness index was calculated as the root-mean-square value of the second principal component score, corresponding to the orthogonal distance from the principal axis (Fig. 4). A smaller flatness index value indicated greater flatness.

**Fig. 4.**
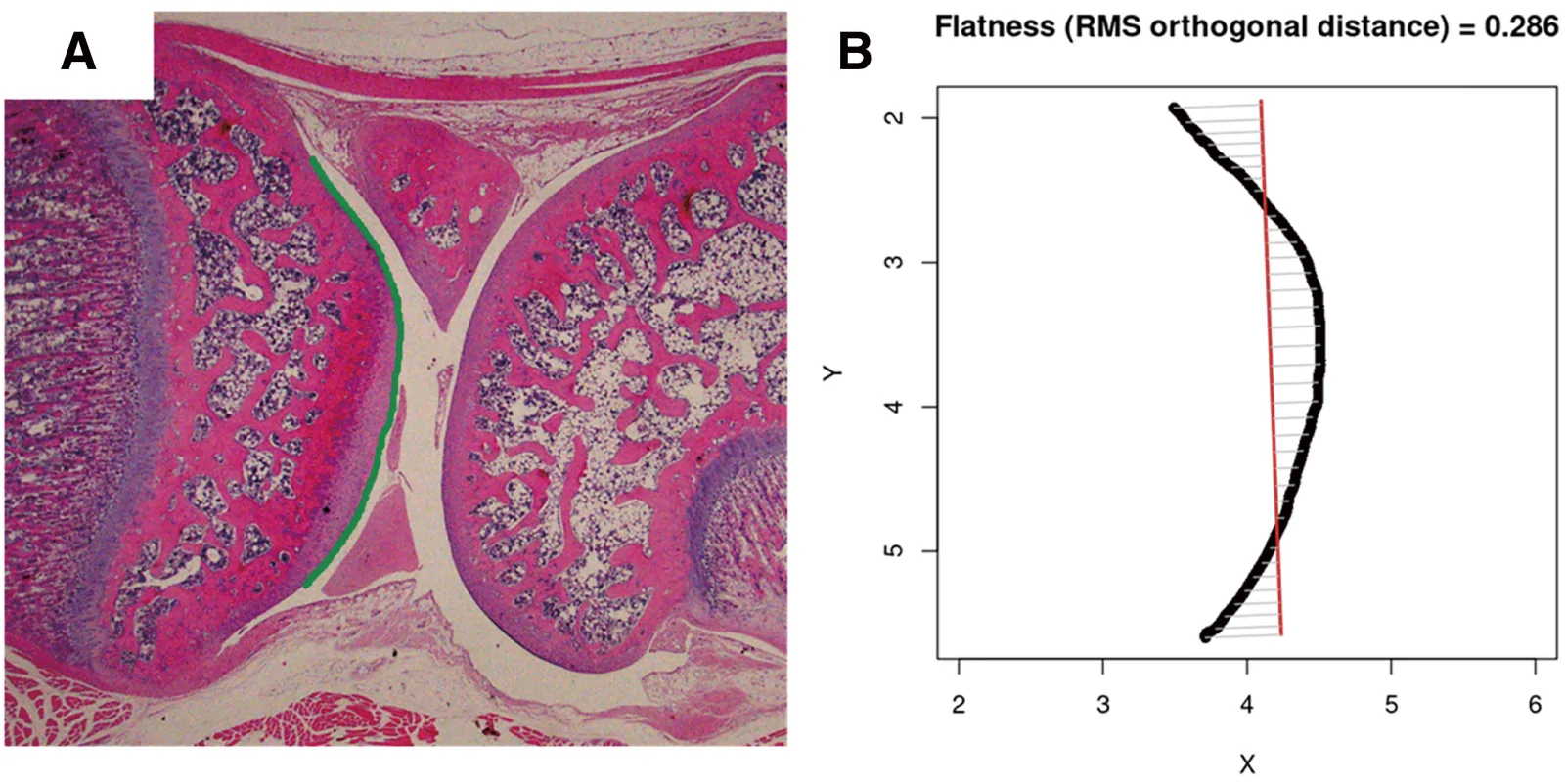
Measurement of the flatness of the tibial cartilage surface. (**A**) Using images acquired at ×20 magnification, the tibial articular surface between the anterior and posterior menisci was selected as the region of interest (green lines), and the XY coordinates were obtained. (**B**) The black dots represent the XY coordinates of the articular cartilage surface. The red line indicates the principal axis obtained by principal component analysis. The gray segments represent the orthogonal distances from each point to the principal axis. Flatness was quantified as the RMS of these orthogonal distances. RMS, root mean square

Comparisons of knee extension angle, posterior joint capsule length, and tibial articular surface flatness were performed using a 2-way analysis of variance with immobilization status and age (duration of immobilization) as factors. Multiple comparisons were performed when significant differences were detected. All statistical analyses were performed using R version 4.0.2 for Windows (CRAN, freeware), with a significance level set at 5%.

## Results

### Knee extension angle and torque curve

The knee extension angles at 10 mNm (the toe region) were 162.7° ± 1.4°, 158.5° ± 2.5°, and 152.9° ± 2.3° in the Con4W, Con8W, and Con16W groups, respectively, and 136.8° ± 3.4°, 118.7° ± 3.9°, and 91.8° ± 14.2° in the Im4W, Im8W, and Im16W groups, respectively. The knee extension angles at the final 120 mNm were 189.2° ± 2.3°, 182.5° ± 2.5°, and 172.8° ± 1.9° in the Con4W, Con8W, and Con16W groups, respectively, and 180.7° ± 2.6°, 161.1° ± 3.8°, and 150.2° ± 5.7° in the Im4W, Im8W, and Im16W groups, respectively. The immobilized groups showed significantly lower values than the age-matched control groups, and this result was consistent with that observed at the other 2 torque values (30 and 60 mNm). Significant differences were observed between the Im4W, Im8W, and Im16W groups at all torque values, with longer immobilization periods resulting in lower values. Similarly, in comparison within the control groups, significant differences were observed among the Con4W, Con8W, and Con16W groups at all torque values, with knee extension angles decreasing with age (Table 1 and the Appendix).

**Table 1.** Knee extension angle at each torque (°)

	Immobilized groups			Control groups		
	4W	8W	16W	4W	8W	16W
10 mNm	136.8 ± 3.4^*,†,‡^	118.7 ± 3.9^*,†^	91.8 ± 14.2^*^	162.7 ± 1.4^†,‡^	158.5 ± 2.5^†^	152.9 ± 2.3
30 mNm	150.5 ± 2.6^*,†,‡^	133.3 ± 2.2^*,†^	116.5 ± 10.7^*^	170.7 ± 3.0^†,‡^	162.7 ± 1.7^†^	158.4 ± 1.4
60 mNm	160.3 ± 3.2^*,†,‡^	143.4 ± 2.8^*,†^	131.7 ± 6.7^*^	177.9 ± 3.0^†,‡^	172.0 ± 2.9^†^	163.7 ± 1.1
120 mNm	180.7 ± 2.6^*,†,‡^	161.1 ± 3.8^*,†^	150.2 ± 5.7^*^	189.2 ± 2.3^†,‡^	182.5 ± 2.5^†^	172.8 ± 1.9

Values were presented as mean ± SD.

^*^Significant difference compared to the age-matched control group, *P* <0.05.

^†^Significant difference from 16W within the same group, *P* <0.05.

^‡^Significant difference from 8W within the same group, *P* <0.05.

W, week; SD, standard deviation

The torque curves in the control groups generally exhibited similar shapes. However, the Con16W group had a smaller final extension angle than the other 2 groups, resulting in a significantly steeper slope in the linear region. The immobilized groups showed increased resistance from the early stages of exercise, with a markedly shortened toe region followed by a gradual linear region, which was different from the torque curve shape of the control groups. The slope of the linear region was significantly different from that of the age-matched control groups, with the Im16W group, which had the longest immobilization period, exhibiting the lowest values. The control groups exhibited strong resistance (indicated by a high slope of the linear region) at the end of the ROM. However, the immobilized groups showed stiffness from the early stage, followed by a gradual resistance (indicated by a low slope of the linear region) that continued thereafter (Fig. 5, Table 2, and the Appendix).

**Fig. 5.**
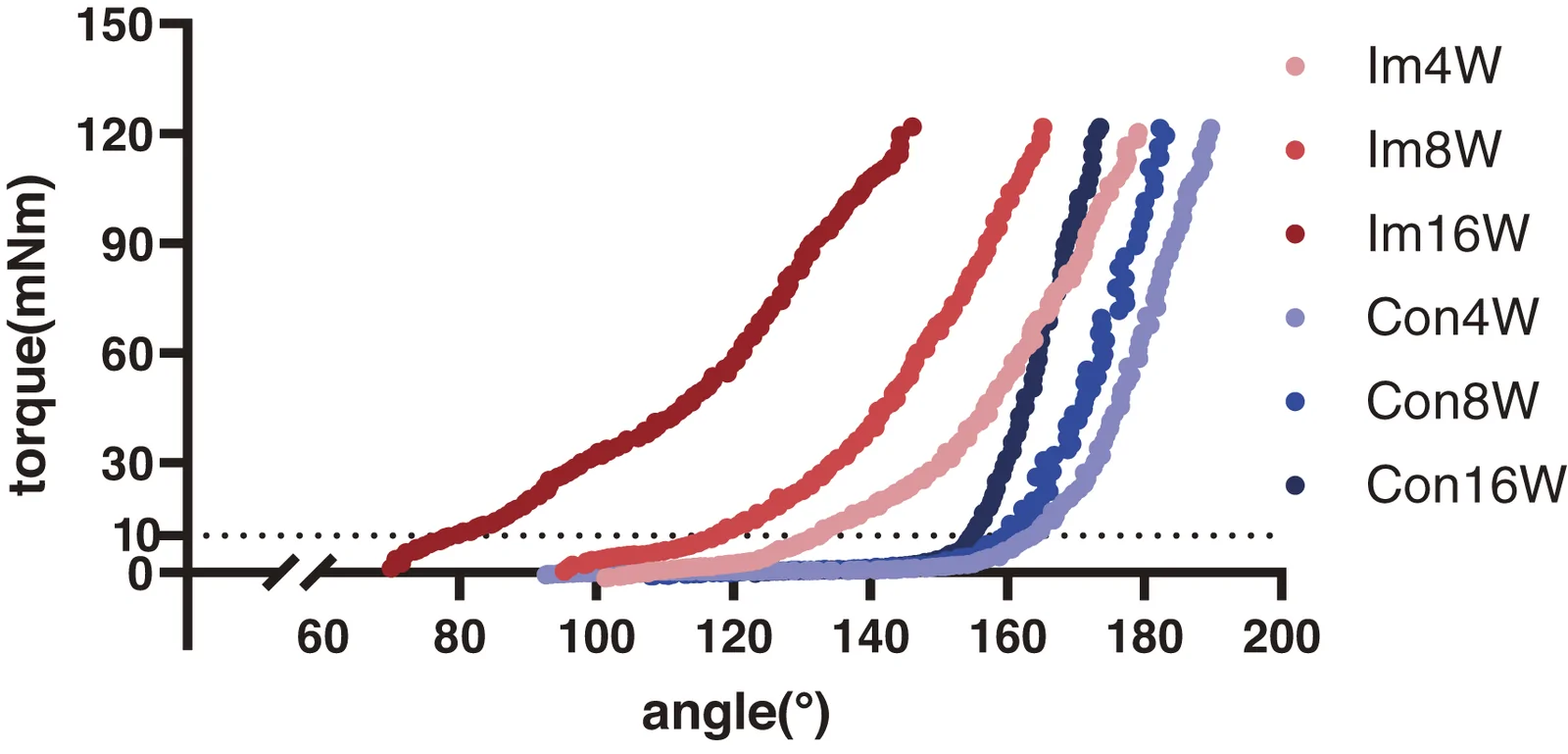
Typical examples of torque curves for each group. In the control groups, the toe region was long in all cases, extending to approximately 150°, followed by a sharp increase in torque values, resulting in a steep slope in the linear region. Conversely, in the immobilization groups, the toe region became shorter as the immobilization period lengthened, followed by a gradual increase in torque values, resulting in a gentler slope in the linear region. Im4W, the 4-week immobilization; Im8W, the 8-week immobilization; Im16W, the 16-week immobilization; Con4W, the 4-week control; Con8W, the 8-week control; Con16W, the 16-week control

**Table 2.** Slope of approximate line in linear region

	Immobilized groups			Control groups		
	4W	8W	16W	4W	8W	16W
ΔNm/Δ°	2.6 ± 0.3^*,†^	2.8 ± 0.3^*,†^	1.9 ± 0.5^*^	4.3 ± 0.3^†^	4.7 ± 0.3^†^	5.8 ± 0.5

Values were presented as mean ±SD.

^*^Significant difference compared to the age-matched control group, *P* <0.05.

^†^Significant difference from 16W within the same group, *P* <0.05.

^‡^Significant difference from 8W within the same group, *P* <0.05.

W, week; SD, standard deviation

### Histological findings

Shortening of the posterior joint capsule was observed in the immobilized groups compared with the control groups. No significant differences were observed within the control or immobilized groups. Thus, no differences were observed based on age or immobilization duration. No inflammatory findings or marked fibrosis were observed in any of the immobilized groups (Fig. 6). Flattening of the superficial layer was observed in the articular cartilage of the immobilized groups. The immobilized groups showed significantly lower degrees of flattening of the tibial articular surface than the control group. However, no differences were observed based on age or immobilization duration (Fig. 7 and the Appendix).

**Fig. 6.**
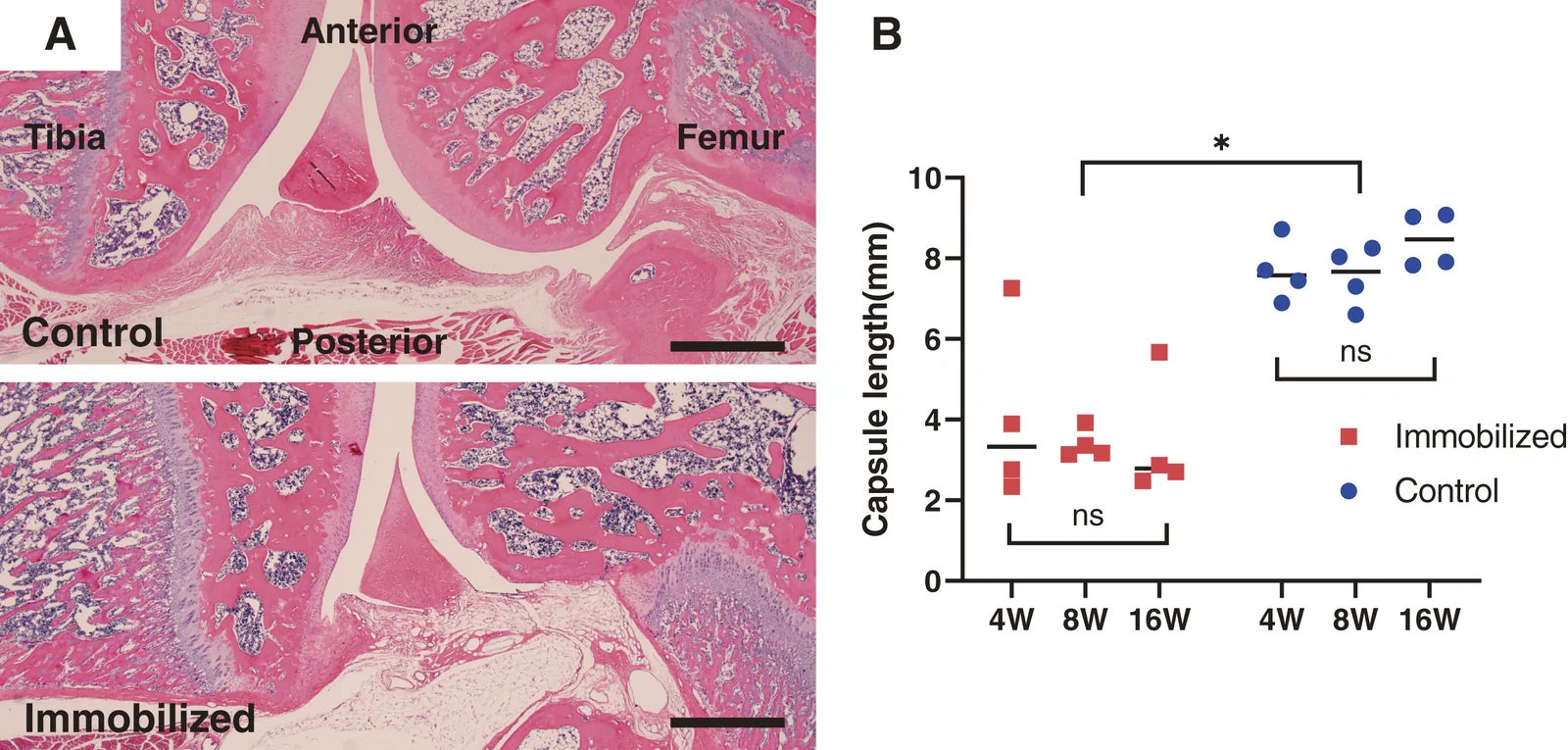
Findings of the posterior knee joint capsule. (**A**) Shortening of the posterior joint capsule was observed in the immobilized groups compared with the control groups. No inflammatory findings or marked fibrosis were observed. H&E staining, scale bar 500 μm. (**B**) Posterior capsular length of the knee joint at 4, 8, and 16 weeks after immobilization, along with the values for the age-matched control groups at each time point. Significant differences were observed between the immobilization and control groups. However, no significant differences were observed within either group. The line indicates the median. *Significant difference compared with age-matched control groups, *P* <0.05. ns indicates no significant difference within the groups (*P* >0.05). H&E, hematoxylin and eosin; W, week

**Fig. 7.**
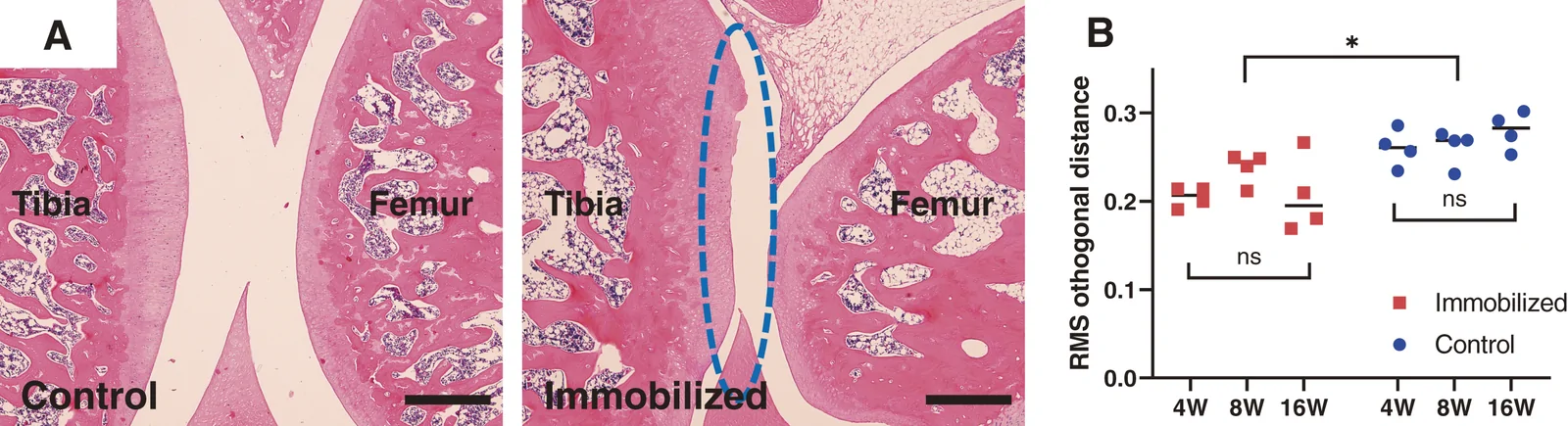
Findings of the articular surface. (**A**) Flattening of the articular surface was observed in the immobilized groups compared with the control groups (dotted circle area). H&E staining, scale bar 500 μm. (**B**) Flatness of the articular surface. Values represent the root mean square of orthogonal distance from the principal axis obtained by principal component analysis for each group. Significant differences were observed between the immobilization and control groups. However, no significant differences were observed within either group. The line indicates the median. *Significant difference compared with age-matched control groups, *P* <0.05. ns indicates no significant difference within the groups (*P* >0.05). H&E, hematoxylin and eosin; W, week

## Discussion

This study showed that, with increasing immobilization period, the torque curve showed a decrease in the toe region and flattening of the slope in the linear region, accompanied by a leftward shift of the torque curve to the left and a change in its shape. Although a steeper slope, indicating greater force for displacement, generally indicates greater stiffness, the shortness of the toe region, which reflects how early resistance to movement reaches a certain threshold, is a more suitable indicator of the stiffness of contracture joints encountered clinically than the slope of the linear region.

Lake et al.^22)^ investigated changes in torque curves using a rat elbow joint model in which the elbow joints were traumatized and immobilized for 6 weeks. They measured ROM and torque during elbow flexion and extension and reported stiffness (N/mm) across 3 zones: the end range of flexion, the end range of extension, and the neutral zone between them. They reported that immobilization reduced ROM and shortened the neutral zone, while increasing stiffness in the neutral zone. However, no change in stiffness was observed at the end ranges of flexion and extension. Their neutral zone likely corresponds to the toe region in our study. The shortening of the neutral zone and the reduction in ROM yielded results similar to those of our study. However, the change in slope at the end range showed different outcomes. This discrepancy may be due to differences in the joints used, the immobilization model employed, and whether the surrounding muscles were included (as they did not excise the muscles before measurement). However, the primary cause is considered to be the relatively low maximum torque applied in their measurements (11.5 mNm), which resulted in the assessment of a different end-range ROM compared with the present study. The leftward shift of the torque curve and the change in its shape likely reflect not merely a shortening of the tissues restricting ROM, which contributes to increased torque, but also qualitative changes in these tissues. Joint immobilization causes fibrosis of the joint capsule^6–9)^ and an increase in immature/abnormal collagen cross-linking^23–25)^. An increase in nonphysiological cross-linking, such as that represented by pentosidine, restricts movement between collagen fibers and increases their stiffness^26–29)^. Therefore, in this study, such changes may have caused early resistance, replacing the region that would normally be the toe region with the linear region. Additionally, the absence of joint capsule shortening proportional to the immobilization period observed in this study supports the notion that qualitative tissue changes, not just simple length changes, influence the torque curve. Unlike length changes, qualitative changes may progress over the long term beyond 4 weeks. Future studies combining the quantification of nonphysiological cross-linking are needed to clarify its relationship to the toe region’s range.

However, investigating the influence of factors other than the joint capsule is necessary. Chimoto et al.^30)^ investigated the effect of the joint capsule on ROM using a contracture model similar to that used in this study by comparing ROM before and after posterior joint capsule incision. They reported that the increase in ROM following joint capsule incision plateaued after 8 weeks of immobilization. Similar findings have also been reported in other studies^11,18,31)^. These findings indicate that factors other than the joint capsule contribute to the torque curve. Additionally, the flattening of the articular surface observed in this study showed no changes corresponding to the immobilization duration and could not be said to directly affect changes in the torque curve. Although immobilization has been reported to increase irregularities in the superficial layer of the cartilage^32)^, no descriptions regarding flattening have been reported. Given that longitudinal bone growth occurs even during immobilization^33)^, this may have caused articular surface compression. This could be clarified in future comparisons with immobilization models using sufficiently aged animals. Similar to the joint capsule, the effects of qualitative changes on cartilage should be considered. Previous studies have reported changes in cartilage microstructure due to immobilization^34)^ and functional decline^35)^, and these changes may have influenced the torque curve regardless of flattening. However, verification based solely on cartilage is difficult. Therefore, changes in ligaments, which are soft tissues connecting the joint beyond the joint capsule, and their impact on joint movement should be examined first. Although ligaments undergo histological and mechanical changes due to immobilization^36–39)^, their contribution to ROM and torque curves remains unclear. The extent to which changes in the torque curve can be induced by sectioning ligaments should be clarified, including sectioning of the medial collateral ligament, which contributes to knee joint stability, and the meniscofemoral ligament, which is known to resist femoral headward displacement by influencing contact pressure and the contact area between the femur and tibia^40,41)^.

The present findings demonstrate that, during the progression of contracture induced by joint immobilization, quantitative limitations in ROM are accompanied by qualitative changes in resistance during joint movement. These findings highlight the need for careful risk management to prevent tissue injury during interventions for joint contracture and provide a pathophysiological basis for the optimization of treatment strategies, including the selection of appropriate loading conditions and therapeutic approaches according to the duration of immobilization. However, this study has some limitations. The small number of specimens used for histological analysis may have limited the ability to detect posterior joint capsule shortening corresponding to the duration of immobilization, as reported in previous studies^8)^. In addition, because qualitative indicators affecting the mechanical properties of the joint capsule were not quantified, the validity of this model as an immobilization model remains insufficiently verified. Further investigation is required, including consideration of the involvement of joint structures other than the capsule. Additionally, the reversibility of the observed torque curve changes following contracture treatment should be clarified in future studies.

## Conclusions

The torque curve of a contracture joint due to joint immobilization does not simply reflect a narrowed version of the torque curve of a normal joint; rather, its shape changes. Specifically, the toe region shortens and transitions earlier into the linear region, whose slope becomes gentler. These changes become more pronounced with prolonged immobilization. These findings indicate that the observed changes are not solely attributable to proportional shortening of the joint capsule but also reflect qualitative alterations in tissue properties.

## Supplementary Materials

Appendix 1. Detail results of analysis of Two-Way ANOVA.
