# Supplementary material for: Changes in Arthrogenic Stiffness Associated with Prolonged Joint Immobilization
Source: Phys Ther Res. 2026 Jun 20;29(2):150–7. doi: 10.1298/ptr.26-E00007 (PMC13624568; doi:10.1298/ptr.26-E00007)
Supplement: Supplementary file 1 [file ptr-29-150-s001.pdf]

Appendix 1. Detailed Results of Two-Way ANOVA

Abbreviations: CON, control group; IM, immobilized group; CI, confidence interval; η^2_p, partial eta squared.

Supplementary Table 1. Summary of two-way ANOVA results

Variable	Group p-value	η^2_p	Age p-value	η^2_p	Group × Age p-value	η^2_p
Knee extension angle at 10 mNm	<0.001	0.938	<0.001	0.806	<0.001	0.633
Knee extension angle at 30 mNm	<0.001	0.931	<0.001	0.836	<0.001	0.529
Knee extension angle at 60 mNm	<0.001	0.943	<0.001	0.878	<0.001	0.472
Knee extension angle at 120 mNm	<0.001	0.893	<0.001	0.907	<0.001	0.525
Slope at linear region	<0.001	0.925	0.095	0.155	<0.001	0.668
Joint capsule length	<0.001	0.802	0.711	0.037	0.523	0.07
Flatness of articular surface	<0.001	0.588	0.406	0.095	0.152	0.189

Supplementary Table 2. Post hoc multiple comparisons for variables with significant Group × Age interaction

Knee extension angle at 10 mNm

Comparison	p-value	95% CI	Result
CON4W vs IM4W	<0.001	22.53 – 29.35	CON > IM
CON8W vs IM8W	<0.001	35.55 – 44.02	CON > IM
CON16W vs IM16W	<0.001	47.97 – 74.27	CON > IM
CON4W vs CON8W	0.010	-7.41 – -1.03	CON4W > CON8W
CON4W vs CON16W	<0.001	-12.97 – -6.59	CON4W > CON16W
CON8W vs CON16W	0.001	-8.76 – -2.38	CON8W > CON16W
IM4W vs IM8W	0.010	-31.55 – -4.57	IM4W > IM8W
IM4W vs IM16W	<0.001	-59.05 – -30.87	IM4W > IM16W
IM8W vs IM16W	<0.001	-40.39 – -13.41	IM8W > IM16W

Knee extension angle at 30 mNm

Comparison	p-value	95% CI	Result
CON4W vs IM4W	<0.001	16.32 – 23.98	CON > IM
CON8W vs IM8W	<0.001	26.87 – 31.87	CON > IM
CON16W vs IM16W	<0.001	31.94 – 51.68	CON > IM
CON4W vs CON8W	<0.001	-11.22 – -4.78	CON4W > CON8W
CON4W vs CON16W	<0.001	-15.52 – -9.08	CON4W > CON16W
CON8W vs CON16W	0.009	-7.52 – -1.08	CON8W > CON16W
IM4W vs IM8W	0.001	-27.19 – -7.24	IM4W > IM8W
IM4W vs IM16W	<0.001	-44.38 – -23.54	IM4W > IM16W
IM8W vs IM16W	0.002	-26.72 – -6.77	IM8W > IM16W

Knee extension angle at 60 mNm

Comparison	p-value	95% CI	Result
CON4W vs IM4W	<0.001	13.38 – 21.78	CON > IM
CON8W vs IM8W	<0.001	25.02 – 32.29	CON > IM
CON16W vs IM16W	<0.001	25.77 – 38.19	CON > IM
CON4W vs CON8W	0.002	-9.62 – -2.18	CON4W > CON8W
CON4W vs CON16W	<0.001	-17.97 – -10.53	CON4W > CON16W
CON8W vs CON16W	<0.001	-12.07 – -4.63	CON8W > CON16W
IM4W vs IM8W	<0.001	-24.08 – -9.86	IM4W > IM8W
IM4W vs IM16W	<0.001	-36.08 – -21.22	IM4W > IM16W
IM8W vs IM16W	0.002	-18.79 – -4.57	IM8W > IM16W

Knee extension angle at 120 mNm

Comparison	p-value	95% CI	Result
CON4W vs IM4W	<0.001	5.07 – 11.75	CON > IM
CON8W vs IM8W	<0.001	17.19 – 25.48	CON > IM
CON16W vs IM16W	<0.001	16.97 – 28.18	CON > IM
CON4W vs CON8W	<0.001	-10.09 – -3.28	CON4W > CON8W
CON4W vs CON16W	<0.001	-19.74 – -12.93	CON4W > CON16W
CON8W vs CON16W	<0.001	-13.05 – -6.25	CON8W > CON16W
IM4W vs IM8W	<0.001	-26.34 – -12.87	IM4W > IM8W
IM4W vs IM16W	<0.001	-37.53 – -23.47	IM4W > IM16W
IM8W vs IM16W	0.002	-17.62 – -4.16	IM8W > IM16W

Slope at linear region

Comparison	p-value	95% CI	Result
CON4W vs IM4W	<0.001	1.26 – 2.14	CON > IM
CON8W vs IM8W	<0.001	1.42 – 2.27	CON > IM
CON16W vs IM16W	<0.001	3.24 – 4.61	CON > IM
CON4W vs CON8W	0.288	-0.23 – 0.93	CON4W = CON8W
CON4W vs CON16W	<0.001	0.89 – 2.05	CON4W < CON16W
CON8W vs CON16W	<0.001	0.54 – 1.70	CON8W < CON16W
IM4W vs IM8W	0.683	-0.43 – 0.83	IM4W = IM8W
IM4W vs IM16W	0.002	-1.14 – -0.10	IM4W > IM16W
IM8W vs IM16W	0.004	-1.58 – -0.33	IM8W > IM16W

Supplementary Table 3. Post hoc comparisons for variables without significant interaction

Joint capsule length

Comparison	p-value	95% CI	Result
CON vs IM	<0.001	-5.23 – -3.25	CON > IM
4W vs 8W	0.782	-1.93 – 1.12	4W = 8W
4W vs 16W	0.993	-1.45 – 1.59	4W = 16W
8W vs 16W	0.717	-1.05 – 1.99	8W = 16W

Flatness of articular surface

Comparison	p-value	95% CI	Result
CON vs IM	<0.001	-0.07 – -0.05	CON > IM
4W vs 8W	0.412	-0.02 – 0.05	4W = 8W
4W vs 16W	0.691	-0.02 – 0.04	4W = 16W
8W vs 16W	0.889	-0.04 – 0.03	8W = 16W
